# The complete chloroplast genome of *Artemisia Montana* (Nakai) Pamp. (Asteraceae), a traditional medicinal herb in Korea

**DOI:** 10.1080/23802359.2021.2002215

**Published:** 2021-11-19

**Authors:** Jung Ju Lee, Yonguk Kim, Dool-Ri Oh, Yujin Kim, Kyo-Nyeo Oh, Donghyuck Bae, Hak-Sung Lee, Dong-Jun Lee, Dong-Wook Kim

**Affiliations:** a Jindo-gun, Jindo County, Republic of Korea; b Jeonnam Institute of Natural Resources Research, Jangheung-gun, Republic of Korea; c Genomics Division, National Institute of Agricultural Sciences(NAS), Jeonju, South Korea; d Coscience Co. Ltd., Muan-gun, Republic of Korea

**Keywords:** Chloroplast genome, *Artemisia montana*, phylogenetic analysis

## Abstract

*Artemisia Montana* (Nakai) Pamp. is a widely used heath food and a well-known traditional Korean herbal medicine. The complete chloroplast genome sequence of *A. Montana* was determined using high-throughput sequencing technology. Chloroplast genome was 151,133 bp in length, with a large single-copy (LSC) region of 98,497 bp, a small single-copy (SSC) region of 18,352 bp, separated by two inverted repeat (IR) regions of 17,142 bp each. It contained a total of 113 genes, with an overall GC content of 37.5%. The phylogenetic analysis showed that *A. montana* most closely related to *A. feddei*. This result will enrich the genetic resources of medicinal plant and useful for future investigation of genetics, evolution and identification of *Artemisia* species.

*Artemisia montana* (Nakai) Pamp. is a perennial herb commonly known as wormwood belong to the family of Asteraceae and widely distributed in Mongolia, China, Korea, and Japan. The *Artemisia* species has been used for many centuries in traditional medicine for the treatment of febrile diseases and malaria in Asia (Yu and Zhong 2001). The artemisinin as its main component is associated with many health benefits such as anti-bacterial, anti-fungal, anti-leishmanial, anti-oxidant, anti-tumour, and anti-inflammatory activities (Efferth 2007; Konkimalla et al. 2008; Ferreira et al. 2010). To gain insight into its evolution and facilitate genetic research into *Artemisia*, we characterized the complete chloroplast (cp) genome of *A. montana* based on Illumina sequencing data.

The *A. montana* was collected from the Department of Agricultural Support in Jindo Island, Korea (34°17′47.7″N 126°02'56.4"E) and deposited at the herbarium of the Jeonnam Institute of Natural Resources (JINR), Korea (voucher specimen number JINR00000121). Total genomic DNA was extracted from leaf samples using a modified CTAB method (Allen et al. 2006). The genome library (paired end, PE = 150 bp) was constructed at Coscience Co. Ltd. (Mokpo, South Korea) using the Illumina Nova Seq 6000 platform (Illumina Inc., San Diego, CA, USA). 454 de novo Assembler software and GS Reference mapper v2.6 (Life Science/Roshe, Penzberg, Germany) were used to conduct quality assessment and used for de novo assembly with the cp genome of closely related species *A. montana* (NC_025910) as the reference. Finally, the assembled cp genome was annotated and adjusted manually using the software Geneious v11.0.4. (Kearse et al. 2012). The complete genome of *A. montana* was submitted to GenBank under accession number LC635379.

The size of cp genome of *A. montana* is 151,133 bp in length including a pair of identical IRs (17,142 bp) separated by LSC (98,497 bp) and SSC (18,352 bp) regions. The overall GC content was 37.5%. It encodes a total of 113 genes, including 86 protein-coding genes, 23 tRNA-coding genes, and 4 rRNA-coding genes.

To confirm the phylogenetic location of *A. montana* within the Asteraceae (family Compositae), a total of 19 complete cp genomes of Asteraceae were obtained from GenBank, and aligned using ClustalW from Mega 7.0 (Kumar et al. 2016). *Ajania pacifica* (Nakai) K.Bremer & Humphries was designated as an outgroup. The phylogenetic analysis was performed using the Neighbour-joining (NJ) method in Mega 7.0 with the Kimura 2-parameter model and 500 bootstrap replicates.

Phylogenetic analysis based on the complete cp genomes showed that *A. montana* was most closely related to *A. feddei* (Figure 1).

**Figure 1.**
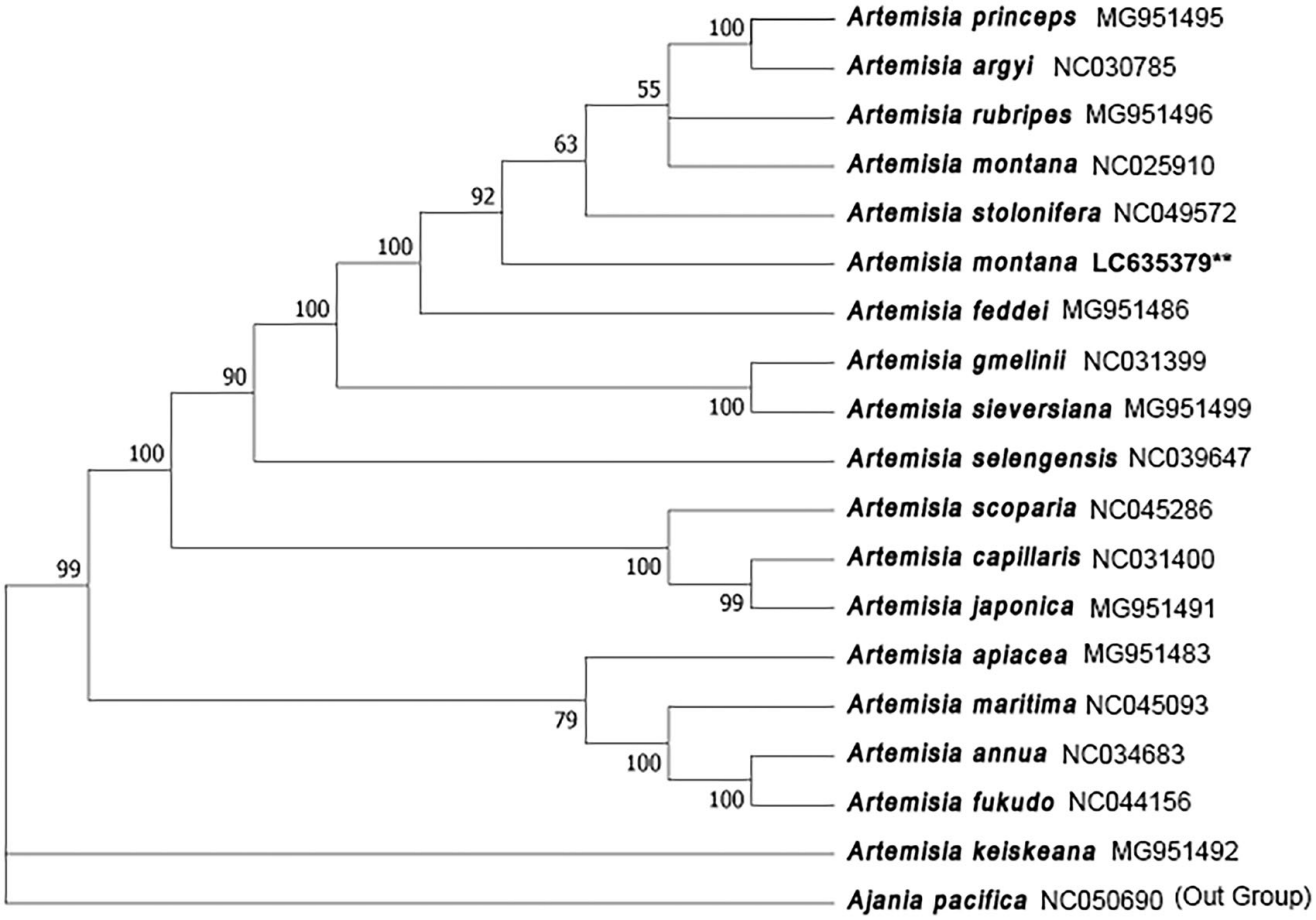
Phylogenetic tree based on 19 complete cp genomes with *Ajania pacifica* as an outgroup. The bootstrap support values (>40%) were shown above the branches. The position of *A. montana* was marked with an asterisk.

## Data Availability

Chloroplast data supporting this study are openly in Genbank at nucleotide database, https://www.ncbi.nlm.nih.gov/nuccore/LC635379.1/, Associated Bioproject, https://www.ncbi.nlm.nih.gov/bioproject/PRJDB11053, Biosample accession number at http://trace.ddbj.nig.ac.jp/BSSearch/biosample?acc=SAMD00327617 and Sequence Read Archive at https://www.ncbi.nlm.nih.gov/sra/?term=DRA011448.
